# NOX4 expression and distal arteriolar remodeling correlate with pulmonary hypertension in COPD

**DOI:** 10.1186/s12890-018-0680-y

**Published:** 2018-07-09

**Authors:** Xiaotong Guo, Yuchun Fan, Jieda Cui, Binwei Hao, Li Zhu, Xiao Sun, Jinxi He, Jiali Yang, Jianda Dong, Yanyang Wang, Xiaoming Liu, Juan Chen

**Affiliations:** 1 Department of Pulmonary and Critical Care Medicine, General Hospital of Ningxia Medical University, 804 Shengli South Street, Xingqing District, Yinchuan, Ningxia 750004 People’s Republic of China; 2 Ningxia Medical University, Yinchuan, 750004 Ningxia China; 3 Department of Radiology, General Hospital of Ningxia Medical University, Yinchuan, 750004 Ningxia China; 4 Department of Thoracic Surgery, General Hospital of Ningxia Medical University, Yinchuan, 750004 Ningxia China; 5 Institute of Human Stem Cell Research, General Hospital of Ningxia Medical University, Yinchuan, 750004 Ningxia China; 6 Department of Radiotherapy, General Hospital of Ningxia Medical University, Yinchuan, 750004 Ningxia China; 7 Department of Pathology, Ningxia Medical University, Yinchuan, 750004 Ningxia China

**Keywords:** Pulmonary hypertension, Chronic obstructive pulmonary disease, Cardiac magnetic resonance imaging, Pulmonary arteriolar remodeling, Nicotinamide adenine dinucleotide phosphate oxidase subunit 4

## Abstract

**Background:**

Pulmonary hypertension (PH) in chronic obstructive pulmonary disease (COPD) is suggested as the consequence of emphysematous destruction of vascular bed and hypoxia of pulmonary microenvironment, mechanisms underpinning its pathogenesis however remain elusive. The dysregulated expression of nicotinamide adenine dinucleotide phosphate (NADPH)-oxidases and superoxide generation by pulmonary vasculatures have significant implications in the hypoxia-induced PH.

**Methods:**

In this study, the involvement of NADPH oxidase subunit 4 (NOX4) in pulmonary arteriolar remodeling of PH in COPD was investigated by ascertaining the morphological alteration of pulmonary arteries and pulmonary blood flow using cardiac magnetic resonance imaging (cMRI), and the expression and correlation of NOX4 with pulmonary vascular remodeling and pulmonary functions in COPD lungs.

**Results:**

Results demonstrated that an augmented expression of NOX4 was correlated with the increased volume of pulmonary vascular wall in COPD lung. While the volume of distal pulmonary arteries was inversely correlated with pulmonary functions, despite it was positively associated with the main pulmonary artery distensibility, right ventricular myocardial mass end-systolic and right ventricular myocardial mass end-diastolic in COPD. In addition, an increased malondialdehyde and a decreased superoxide dismutase were observed in sera of COPD patients. Mechanistically, the abundance of NOX4 and production of reactive oxygen species (ROS) in pulmonary artery smooth muscle cells could be dynamically induced by transforming growth factor-beta (TGF-β), which in turn led pulmonary arteriolar remodeling in COPD lungs.

**Conclusion:**

These results suggest that the NOX4-derived ROS production may play a key role in the development of PH in COPD by promoting distal pulmonary vascular remodeling.

## Background

Chronic obstructive pulmonary disease (COPD) is a complex disease characterized by airflow limitation, inflammation and airway remodeling. It is one of the most important causes of death in aging population, and is associated with a significantly economic burden worldwide [1, 2]. Pulmonary hypertension (PH), an important negative prognostic sign may be developed during the late course of COPD [3]. Despite the pathogenesis of PH in COPD remains incompletely understood, the loss of pulmonary capillary bed in emphysema is suggested to contribute to the increased pressure in pulmonary circulation. In this regard, the pulmonary vascular remodeling is widely recognized as a key factor in development of hypoxic pulmonary hypertension (HPH), and a main cause of COPD. It is worthy to note that the process of pulmonary vascular remodeling includes the proliferation and hypertrophy of pulmonary smooth muscle cells and deposition of extracellular matrixes (ECM) [4].

In patients with COPD, the enlargement of pulmonary artery was found to correlate with right ventricular (RV) dysfunction and loss of blood volume in small pulmonary vessels [5], which precedes the development of PH and cor pulmonale. Both of these two manifestations are independent predictors of poor survival [6]. However, controversial results were also reported in several studies, in which vascular changes in COPD were failed to identify any abnormalities, or found only minimal increased volume of pulmonary arterial walls, even in lungs of patients with severe COPD [7, 8]. These conflicting data indicate a complexity of COPD pathogenesis.

It has been well recognized that the oxidative stress is one of the major causes of COPD [9]. An increase of reactive oxygen species (ROS) production is involved in changes of vasoreactivity, endothelial dysfunction, and vascular remodeling including vascular wall cell proliferation and vasoconstriction [9–11]. In this respect, NADPH oxidases (NOXs) have been recognized as important sources of superoxide production in vasculatures [9, 12]. Indeed, the NOX-mediated redox signaling is implicated in vascular remodeling of human pulmonary artery cells and the PAH development [13–15]. Several lines of studies have revealed that the hypoxia-induced expression of NADPH oxidase subunit 4 (NOX4) played a critical role in the development of PH in mice model [13, 16–19]. Clinically, an increased NOX4 was observed in pulmonary vasculatures of both chronic PH and human idiopathic pulmonary arterial hypertension (PAH) [13–15]. Of importance, the NOX4 was a relevant NOX homolog in human airway, which could be induced by transforming growth factor beta (TGF-β) in human PASMCs (HPASMCs) and had an important implication in pulmonary vascular remodeling [12, 19, 20]. Our previous study also demonstrated that an augmented expression of NOX4 and TGF-β was correlated with the increased volume of airway smooth muscle (ASM) mass and epithelial cells of small airways in patients with COPD [21]. Moreover, the increased expression of NOX4 was accompanied by an up-regulated TGF-β in ASM of small airway in COPD lungs [21]. These studies thus strongly imply a contribution of NOX4 and TGF-β in the development of PH in COPD. However, the regulation of NOX4 in peripheral artery smooth muscle cells of COPD patients and its clinical implications in the pathogenesis of PH in COPD has yet been extensively explored.

Cardiac magnetic resonance imaging (cMRI) enables a combination of morphological and functional assessment of right ventricular (RV) and pulmonary circulation, including left ventricular (LV) and RV chamber sizes, wall thickness and mass, and velocity of flow in blood vessels [22]. Therefore it can be harnessed to detect early RV dysfunction and remodeling in patients with non-severe COPD, and used to noninvasively diagnose PH with high accuracy (92%). For instance, the late gadolinium enhancement, retrograde flow ≥0.3 L/ (min⋅m^2^), and pulmonary artery relative area change ≤15% measured by cMRI were useful parameters for predicting the presence of PH with a high degree of diagnostic certainty in patients with COPD [5, 23, 24]. Equally noteworthy, previous data from cMRI measurements have suggested that the right ventricular ejection fraction (RVEF) and right ventricular myocardial mass (RVMM) were correlated with the severity of airflow limitation in COPD [24].

In view of aforementioned findings, we therefore sought to assess the morphological characteristics of distal pulmonary arteries by cMRI measurement, and investigate the relationship between NOX4 of distal pulmonary arteries and airflow limitation in patients with COPD. In order to better understand the role and mechanism underlying NOX4 in distal pulmonary vascular remodeling, relationships between the NOX4 and distal pulmonary artery remodeling, pulmonary circulation and/or ventricular morphology of COPD patients were explored. In addition, the interaction between NOX4 and TGF-β in human primary artery smooth muscle cells (HPASMCs) was also investigated.

## Methods

### Ethics statement

Human samples were collected with a protocol approved by the Ethic Committee for the Conduct of Human Research at Ningxia Medical University (NXMU-2015-205). Written consent was obtained from every individual according to the Ethic Committee for the Conduct of Human Research protocol. All participants were above 18 years old, and were provided a written informed consent for the publication of the data. The Ethic Committee the Conduct of Human Research at General Hospital of Ningxia Medical University approved the consent procedure for this study (NXMU-2015-205).

### Subjects

A total of 15 patients with COPD (ages range 43–64 years) and 19 of gender- and age- matched individuals (ages range 45–68 years) with normal lung function were recruited in General Hospital of Ningxia Medical University between January 2015 and December 2016. All enrolled individuals who were about to undergo pneumonectomy, or lobectomy for suspected early stage of non-small cell lung cancer (NSCLC). Of the 15 enrolled patients in the COPD group, 8 had lung adenocarcinomas, three had squamous lung cancer (among the 11 lung cancer patients, 6 patients suffered TNM stage I and 5 patients were with stageII cancer), and the rest 4 patients had benign lesions, as confirmed by histology. Of all 19 individuals in non-COPD group, 14 had lung adenocarcinomas, four suffered from squamous lung cancer (among these cancer patients, 9 patients were with TNM stage I and 8 patients with stageII cancer), and 2 had benign lesions. The stages of NSCLC were diagnosed according to the NCCN guideline of TNM staging of NSCLC (2016 version) (https://www.nccn.org/).

Basic demographic information was collected using a specifically designed questionnaire after a written informed consent was obtained. Smoking status was defined as nonsmoker (never smoking), ex-smoker (smoking quit for at least 6 months), and current smoker (smoking at least one cigarette daily for more than 6 months). The cigarette consumption was calculated by multiplying the number of pack of cigarette smoked per day by years of smoking (pack-years). Basic data on pulmonary function testing (PFT) [25, 26], echocardiography [27], and 6-min walk distance (6MWD) [28] were also collected (Table 1). All subjects underwent cMRI and echocardiography in General hospital of Ningxia Medical University (Yinchuan, China). Standard pulmonary function testing was performed on all subjects before the surgical performance. The pulmonary function was ascertained by measured the postbronchodilator forced vital capacity (FVC) and forced expiratory volume of one second (FEV1), using a MasterScreen PFT spirometer system (Care Fusion, San Diego, CA, USA). The diagnosis of COPD was essentially according to the criteria of Global Initiative on Obstructive Lung Disease (GOLD 2015) [1]. The exclusive criteria: patients accompanied with 1) other chronic lung diseases, such as bronchial asthma, sleep apnea-hypopnea syndrome, bronchiectasis, pulmonary fibrosis, interstitial lung disease; 2) abnormal liver and kidney function; 3) known ischemic heart disease; 4) congestive heart failure; 5) structural heart disease; 6) PH; 7) prior thromboembolic disease; 8) cerebrovascular disease; 9) peripheral arterial disease; 10) hepatitis and autoimmune diseases and/or 11) inability to undergo cMRI were excluded in this study. According to the guideline of GOLD 2015, the distribution of spirometric classification in 15 recruited patients with COPD was as follow: 6 out of the 15 patients with mild stage of COPD, 9 with moderate stage (patients with mild stage and moderate stage of COPD were grouped in the moderate COPD for statistics in the study). A portion of grossly normal lung tissue with size of approximately 1.0 cm^2^ in area and 0.5 cm of thickness was collected from the distal end of the lesion (≥5.0 cm) during the process of operation. Tissue specimens were harvested after written informed consents for the publication of the data were obtained. The specimen was immediately snap frozen in liquid nitrogen (LN) for protein and RNA analysis, embedded in optimal cutting temperature (OCT) compound, or immerged in 10% buffered formalin fixative.

**Table 1:** Demographics of patients with COPD and non-COPD control subjects

Demographics	Control	Patients with COPD		t/t’	*P*
		Mild (GOLD1)	Moderate (GOLD2)		
Subjects	19	6	9		
Age (s)	55.84±7.82 (45–68)	54.83±7.36 (43–64)	54.11±6.92 (43–64)	0.56	0.58
Gender					
Male	13	6	6	0.74	0.46
Female	6	0	3		
Stature (m)	1.68±0.07	1.70±0.07	1.68±0.05	− 0.45	0.65
Weight (kg)	69.79±11.63	65.33±7.00	55.84±14.38		
BSA (m2)	1.76±0.18	1.72±0.12	1.71±0.22	0.86	0.40
BMI (kg/m^2^)	24.69±2.95	22.75±2.25	22.77±3.56	1.87	0.07
Smoking status and cigarette consumption (Pack-years)	9.66±11.83	19.17±14.97	13.33±15.81	−1.296	0.20
Never smoking (n)	9	1	4		
Current smoking (n)	6	4	3		
Ex-smoker (n)	4	1	2		
PO_2_ (mmHg)	92.51±7.34	60.85±8.36	63.63±2.72	7.82	0.00
PCO_2_ (mmHg)	37.67±2.60	41.20±4.81	41.56±4.53	−2.88	0.01
FEV_1_/FVC (%)	76.29±6.04	63.67±3.56	53.22±8.26	7.30	0.00
FEV_1_%prep (%)	97.74±18.80	91.00±4.47	62.22±4.09	4.02	0.00
DLCO(%)	87.89 ± 8.43	86.00 ± 8.32	67.89 ± 6.49	3.72	0.00
6MWD (m)	507.00±25.27	422.33±49.94	425.89±37.25	6.84	0.00

*6MWD* six-Minute Walk Distance, *BSA* body surface area, *BMI* body mass index, *FEV1/FVC* forced expiratory volume in one second/ forced vital capacity, *FEV1%pred* forced expiratory volume in one second total predicted value, *PCO2* partial pressure of carbon dioxide, *PO2* arterial partial pressure of oxygen. Data was presented as mean ± SD, and *P* values were given as COPD versus non-COPD groups

### Cardiac magnetic resonance imaging (cMRI)

The cMRI examination was performed using a 3.0-T MRI scanner (GE Healthcare, Buckinghamshire, UK,). All cMRI images were acquired using an eight-channel heart array coil, followed by electrocardiogram gating and breath-holding, with the patient in a supine position. All subjects needed to repeat breath exercises before being scanned. The axial, sagittal, and coronal planes were scanned and images were acquired in end-expiratory breath-hold. A BH Ax Fiesta sequence was used to collect 10 layers of axial images in the coronal plane at the center of main pulmonary artery. The Oblique Fiesta and Fast Cine PC sequences were used to obtain images of the main pulmonary artery, while the two-dimensional steady-state precession fast acquisition sequence (Fiesta) was used to obtain LV and RV two-chamber and four-chamber heart and short-axis cMRI images. The RV function was analyzed using Fiesta short-axis images. All images were transferred to the Advantage Windows workstation 4.3 (GE Medical Systems, WI, USA) after scanning. The Report Card 4.0 cardiac function analysis software (GE Healthcare, Milwaukee, WI) was used for measuring parameters of heart function. Two professional radiologists blindly evaluated data independently by measuring the maximum and minimum cross-sectional area (CSA) of main pulmonary artery during the cardiac cycle. The main pulmonary artery distensibility (mPAD%) was calculated using the following equation: mPAD% = (CSAmax-CSAmin)/CSAmin × 100% [29]. The epicardial and endocardial contours of RV of systolic and diastolic short-axis images were manually traced on screen. Functional parameters of heart, such as RVEF and left ventricular ejection fraction (LVEF) were automatically calculated. The myocardial mass was assessed using the following equation: myocardial mass = (epicardial volume - endocardial volume) × 1.05 (specific gravity of myocardium). Then, the right ventricular myocardial mass end-diastolic (RVMED), right ventricular myocardial mass end-systolic (RVMES), and right ventricular mass index (RVMI) were obtained as previously described [30, 31].

### Cell culture and treatment

Human primary artery smooth muscular cells (HPASMCs) were purchased from ScienCell Research Laboratories (Carlsbad, CA, USA) and cultured in SMCM basic medium (ScienCell Research Laboratories, Carlsbad, CA, USA) supplemented with 10% fetal bovine serum (FBS), 100 U/mL penicillin and 100 μg/mL streptomycin at 37 °C in a humidified atmosphere with 5% CO_2_. Cells between passages 3–12 were used in this study. Cells were treated with TGFβ_1_ at various concentrations (0–10 ng/mL) for different time periods (0–48 h).

### Morphometric studies

**Fig. 1:**
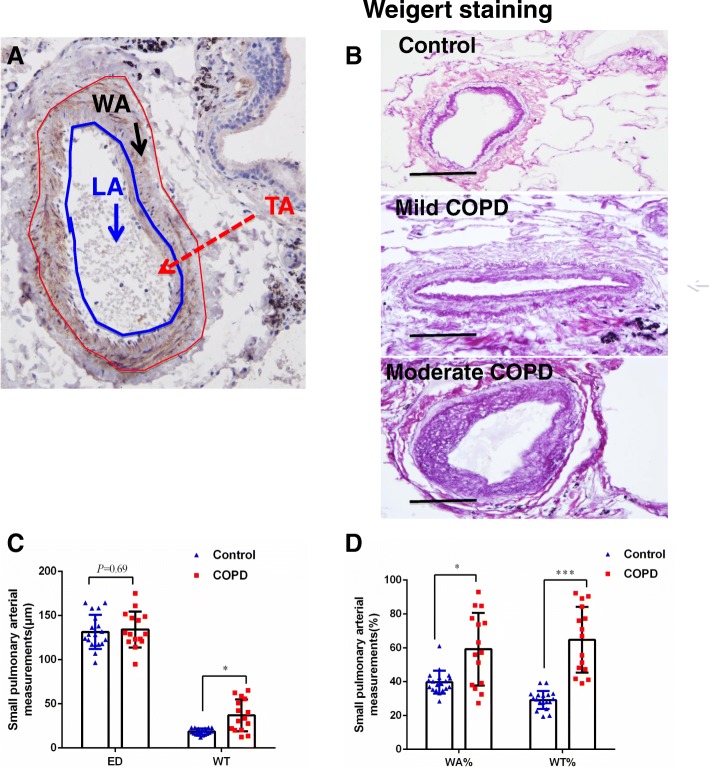
An increased volume of distal pulmonary arteries in COPD patients. **a** The image illustrates the method for measurement of muscular area of pulmonary arteries in this study (× 400). The total area of artery (TA, area inside the red line), luminal area of artery (LA, area inside the blue line), the vascular wall area (WA, the area between red line and blue line) were measured by Image-Pro Plus software 6.0. **b** Weigert-van Gieson staining showed more abundant collagen and connective tissues in arteries of COPD lung relative to controls; (**c**) comparison of external diameter (ED) and vascular diameter (WT) determined by IHC staining of α-SMA between normal and COPD lungs; (**d**) comparison of the pulmonary artery smooth muscle area accounted for the percentage of total vascular area (WA%) and the pulmonary artery smooth muscle thickness accounted for the percentage of vascular diameter (WT%) determined by IHC staining of α-SMA between non-COPD and COPD lungs. Compared to non-COPD lung, *: *p* < 0.05 01 (*N* = 19 for non-COPD; *N* = 15 for COPD)

Lung tissues were embedded in paraffin or optimal cutting temperature (OCT) compound, and cut at a thickness of 4 μm for hematoxylin and eosin (HE) or Weigert-van Gieson staining (to highlight collagen and elastic fibers). Tissue sections from all subjects were stained with HE for histopathologic examination. Pulmonary vascular elastic fibers and collagen were determined on paraffin sections by Weigert-van Gieson staining using a kit (DC0066B7, Leagene, Beijing, China) [32, 33]. The morphometric characteristics of smooth muscle of distal pulmonary arteries were analyzed in sections immunohistochemically stained with α-smooth muscle actin (α-SMA) and Weigert-van Gieson staining (Fig. 1a). Arteries with an external diameter 100–500 μm and completely elastic laminas were evaluated as previously reported [34–36]. 5–10 arteries with an external diameter 100–500 μm per subject were evaluated in this study. The external and internal elastic lamina and the inner area of the intima were outlined; the area of muscular layer, intimal layer, and lumen were computed; and areas were expressed as a percentage of the total measured area. The stained slide was examined using the Olympus light microscope BX51 (Olympus China, Beijing, China), and images were analyzed using Image-Pro Plus 6.0 (IPP6.0) software (Media Cybernetics, MD, USA). The computer-assisted quantification of the staining in a selected area was performed in images with a magnification of 400×. For measurements, the total area of artery (TA), luminal area of artery (LA), and perimeter of blood vessels (P) were measured using IPP6.0 as outlined in Fig. 1a. The following parameters were further calculated: the blood vessel radius (*R*) = *P*/2π; the external diameter of blood vessels (ED) = R + WT/2; the vascular wall area (WA) = TA-LA; and the thickness of blood vessel wall (WT) = WA/*P*. The thickness of pulmonary vessel wall was expressed as the percentage of external diameter calculated by the formula as (2×WT/ED)×100% [4, 37]. The thickness of pulmonary vessel wall accounted for the percentage of vascular diameter (WT%), and the area of pulmonary artery smooth muscle accounted for the percentage of total vascular area (WA%) = WA/TA×100% [38].

### Immunocytochemical histochemical,and immunohistochemical staining

For immunocytochemical staining, HPASMCs were fixed in 4% paraformaldehyde for 15 min and permeabilized with 0.1% Triton X-100 in PBS for 10 min at room temperature. The cells were blocked with 5% bovine serum albumin (BSA; Sigma Chemical Co., MO, USA) for 1 h at room temperature, washed with PBS, and probed with rabbit anti-NOX4 antibody (1:500, Novus Biotech, CO, USA), mouse anti-collagen I antibody (1:500, Abcam, MA, USA), mouse anti-TGFβ1 antibody (1:1000, Abcam, MA, USA) or mouse anti-α-smooth muscle actin (SMA) antibody (1:200, Abcam, MA, USA) overnight at 4°C. Species-matched normal sera were served as negative controls of antibodies. The cells were rinsed in PBS for 3×5 min and incubated with peroxidase-labeled appropriate secondary antibodies (ZSGB-Bio, Beijing, China) (1:1000 in blocking buffer) for 45 min at room temperature. The signal of interest was developed with 3,3′-diaminobenzidine (DAB) peroxidase substrate.

The immunohistochemical analysis was performed on paraffin-embedded tissues as previously described [21]. Briefly, the deparaffined sections were incubated with 0.3% H_2_O_2_ in methanol to inhibit endogenous peroxidase activity, and non-specific binding was blocked by incubating sections with 5% BSA for 1 h at room temperature. The sections were probed with antibodies against proteins of interest as described in immunocytochemical staining, except they were counterstained with hematoxylin if applicable. The stained sections were examined and photographed. 5–10 arteries with an external diameter 100–500 μm per subject were evaluated the average optical density (AOD) of target protein in PASM in lung tissue. Five randomly fields of each section at a magnification of 400× were used for analyzing the positive staining as previously reported [21, 33]. The obtained images were then for a semi-quantitative analysis of the expression of protein of interest by measuring the integrated absorbance (IA) or optical density (OD) using the IPP6.0 software, and the AOD values of each sample were used as an index of the expression of proteins.

### Quantitative reverse transcriptional polymerase chain reaction (qRT-PCR)

HPASMCs grown in six-well plates were rinsed with PBS and lysed directly in culture dished with 1.0 mL of TRIzol reagent (Invitrogen, CA, USA). For lung tissues, 50 μg frozen lung tissues were lysed directly in 1.0 mL of TRIzol reagent. Total RNAs were extracted and 1.0 μg of total RNA was used for reverse-transcription using Oligo (dT) and Transcriptor Reverse Transcriptase (TaKaRa, Japan) according to the manufacturer’s instruction (RNeasy Mini Kit, TaKaRa, Japan). cDNAs were amplified using gene-specific primers from published data or designed using Oligo version 6 (Molecular Biology Insights, CO, USA). Transcripts were amplified by polymerase chain reaction (PCR) using a specific primer for NOX4, α-SMA and glyceraldehyde-3-phosphate dehydrogenase (GAPDH). The quantitative reverse transcriptional PCR (qRT-PCR) was performed using the RT2 SYBR Green qPCR Mastermix (Qiagen, Duesseldorf, Germany) in Lightcycler 480 Real-Time System (Roche, Basel, Switzerland). qRT-PCR was performed with following parameters: 95 °C for 10 min, 45 cycles of 94 °C for 5 s and 60 °C for 30 s, and 60 °C for 5 min followed by a dissociation curve analysis. The expression of transcripts was analyzed using the ^∆∆^CT method [39]. Each sample was examined in triplicate, and the abundance of transcripts was normalized against GAPDH. Primers used in this study were as follows: NOX4 forward: 5’-AGATGTTGGGGCTAGGATTG-3′, reverse: 5’TCTCCTGCTTGGAACCTTCT-3′; α-SMA forward: 5’-GACCGAATGCAGAAGGAGAT-3′, reverse: 5’-CCACCGATCCAGACAGAGTA-3′; GAPDH forward: 5’-CAGCCTCAAGATCATCAGCA-3′, reverse: 5’-ACAGTCTTCTGGGTGGCAGT-3′. TGFβ1 forward: 5’-GAAATTGAGGGCTTTCGCCT-3′, reverse: 5’-AGTGAACCCGTTGATGTCC-3’.

### Western blotting analysis

The frozen lung tissues were stored at − 80 °C and 1.0 ml of ice-cold RIPA buffer containing a protease inhibitor cocktail (Roche, Basel, Switzerland) was added to approximately 300 mg of lung tissue for homogenization. HPMECs were harvested on ice and proteins were extracted with lysis buffer containing a protease inhibitor cocktail as described elsewhere. Protein concentrations were determined by the BCA assay. Proteins were resolved in SDS-PAGE and transferred on nitrocellulose filter membrane, blots were probed for NOX4, TGFβ1, collagen, α-SMA or GAPDH with appropriate antibodies, respectively. Protein of interest was detected using a horseradish peroxidase (HRP)-labeled secondary antibody (ZSGB-Bio Ltd., Beijing, China) and acquired with enhanced chemiluminescence (ECL) (Thermo Fisher Scientific, MA, USA). The protein expression levels were quantified by optical densitometry using NIH ImageJ Fiji Software (https://imagej.net/Fiji) if applicable. Fold change was calculated as the ratio between the net intensity of each sample divided by the respective internal controls (GADPH) as previously described elsewhere [40].

### Measurement of malondialdehyde (MDA), superoxide dismutase (SOD) and ROS

The concentration of MDA and SOD in HPASMC were measured using commercial assay kits (E-EL-0060c for MDA and E-EL-H2382c for SOD, Elabscience, China) according to manufacturer’s protocols. The concentration of Collagen Type 1 alpha1 in HPASMC culture supernatants was measured using commercial assay kits (ab210966, Abcam, MA, USA) according to manufacturer’s protocols. Intracellular ROS level was determined by accessing the mean fluorescent intensity of 2′-7′-dichlorodihydrofluorescein diacetate (H_2_DCFH-DA, Molecular Probes, OR, USA) using a flow cytometric assay with 495-nm excitation and 525-nm emission (BD Biosciences, CA, USA). Briefly, HPASMCs were seeded in six-well plates and cultured for 24 h in SMCM media before they were exposed to media containing different concentrations (0–10 ng/mL) of TGFβ_1_ for various time periods (0-48 h). For measurement of MDA and SOD in HPASMC, cells washed twice with cold PBS, then added the protein lysis (RIPA with proteinase inhibitor) on the ice, scraped and collected the lysis, centrifuge at 12000 rpm for 20 min. The supernatant was collected and sample protein concentration in the extract was quantified using a BCA protein assay. The protein expression of MDA and SOD was determined in supernatant of cell lysate using an ELISA kit. For measurement of ROS in HPASMC, the cells were rinsed with warm PBS and incubated in serum-free and phenol red-free DMEM/F-12 medium containing 10μΜ H_2_DCFH-DA at 37°C for 30 min. At the end of incubation, the medium was removed, and the cells were rinsed twice with pre-warm PBS prior to intracellular ROS analysis using a flow cytometer.

### Statistical analysis

All data were expressed as mean ± standard error of the mean (SEM). The independent-samples *t* test (for two-group comparison) and one-way analysis of variance (for multiple-group comparison), and least significant difference test were employed to compare between groups using SPSS 19.0 software (Chicago, IL, USA). The Pearson correlation analysis was used to analyze correlations between the pulmonary artery smooth muscle thickness accounting for the percentage of vascular diameter (WT%), the pulmonary artery smooth muscle area accounting for the percentage of total vascular area (WA%), FEV1/FVC, FEV1%pred, and the abundance of NOX4 protein in the pulmonary arteriolar smooth muscles. A *P* value less than 0.05 was considered as a statistical significance.

## Results

### Demographic data

Fifteen patients with COPD were enrolled in this study, included 3 females and 12 males with a mean age of 54.40 ± 6.84 years (range of 43–64 years). Of these, six were diagnosed with mild COPD (all males; four current smokers, one ex-smoker, and one never smoker) and nine with moderate COPD (six males and three females; three current smokers, two ex-smokers, and four never smokers). The cigarette consumption (pack-years) in the COPD group was 15.67 ± 15.22; of enrolled patients in the COPD group, eight had lung adenocarcinomas, three had squamous lung cancer, and four had benign lesions confirmed by histology. Nineteen non-COPD patients were enrolled in this study, included six females and thirteen males with a mean age of 55.84 ± 7.82 years (range of 45–68 years). Six non-COPD patients were current smokers, four were ex-smokers, and nine were never smokers. The cigarette consumption (pack-years) in non-COPD group was 9.66 ± 11.83. Of all individuals in non-COPD group, thirteen had lung adenocarcinomas, four suffered from squamous lung cancer, and two had benign lesions, as confirmed by histology. No significant difference in age, body mass index (BMI), cigarette consumption (pack-years), and tumor histology was found between patients with COPD and without COPD. But parameters of pulmonary functions FEV1%pred, FEV1/FVC%, and 6MWD were significantly different in patients with COPD compared with non-COPD subjects (Table 1).

### Parameters of ventricular dimensions and pulmonary circulation on cMRI

cMRI images showed an enlargement of main pulmonary artery and a reduced left and right ventricle in patients with COPD compared to those without COPD (Fig. 1b). The cMRI parameters of mPAP (22.78 ± 1.6 vs 24.98 ± 3.68, *P* = 0.02), mPADmax (26.96 ± 2.13 vs 30.36 ± 3.34, *P* = 0.00), mPADmin (22.06 ± 2.15 vs 24.91 ± 2.50, *P* = 0.00), CSAmax (5.77 ± 0.99 vs 6.98 ± 0.95, *P* = 0.00), and CSAmin (3.69 ± 0.69 vs 4.91 ± 0.98, *P* = 0.00) exhibited statistical difference between patients with COPD and those without COPD, respectively (Table 2). The average negative flow (ANF) (3.25 ± 2.00 vs 5.27 ± 2.55, *P* = 0.01) and regurgitant fraction (RF%) (4.68 ± 3.04 vs 7.91 ± 3.74, *P* = 0.01) also showed statistically different (*P* < 0.05), despite the positive peak velocity, negative peak velocity, average volume flow, and average positive flow were not statistically different between COPD and non-COPD groups (*P >* 0.05). In addition, cMRI parameters of ventricular dimensions in individuals with COPD and without COPD, including RVMED (32.94 ± 4.31 vs 40.03 ± 8.55, *P* = 0.01), RVMES (28.05 ± 5.00 vs 32.87 ± 8.10, *P* = 0.04), and RVMI (0.38 ± 0.08 vs 0.46 ± 0.13, *P* = 0.04) also displayed a statistical difference (*P* < 0.05), respectively. But the RVEDV, RVESV, RVSV, and RVEF were not statistically different between these two groups (*P >* 0.05) (Table 2).

**Table 2:** Cardiac magnetic measurement parameters

	Control (*n* = 19)	COPD (*n* = 15)	*t/t’*	*P*
mPAP (mmHg)	22.78±1.62	24.98±3.68	−1.40	0.02
mPADmax (mm)	26.96±2.13	30.36±3.34	−3.61	0.00
mPADmin (mm)	22.06±2.15	24.91±2.50	−3.59	0.00
CSAmax (cm2)	5.77±0.99	6.98±0.95	−3.60	0.00
CSAmin (cm2)	3.69±0.69	4.91±0.98	−4.26	0.00
mPAD (%)	57.29±14.47	43.82±10.87	2.99	0.01
PPV (cm/s)	69.71±15.76	74.71±26.79	−0.68	0.50
PNV (cm/s)	21.42±14.28	27.31±14.81	−1.18	0.25
AF (ml/beat)	70.01±22.00	64.73±17.31	0.76	0.45
APF (ml/beat)	72.93±22.27	68.50±16.75	0.64	0.53
ANF (ml/beat)	3.25±2.00	5.27±2.55	−2.60	0.01
RF (%)	4.68±3.04	7.91±3.74	−2.78	0.01
RVEDV (ml)	124.28±21.53	122.15±25.78	0.26	0.80
REESV (ml)	58.51±14.57	58.63±22.03	−0.02	0.99
RVSV (ml)	65.77±16.68	63.53±13.99	0.42	0.68
RVEF (%)	52.81±10.19	51.27±8.97	0.46	0.65
RVMED (g)	32.94±4.31	40.03±8.55	−2.93	0.00
RVMES (g)	28.05±5.00	32.87±8.10	−2.132	0.04
RVMI (g/m2)	0.38±0.08	0.46±0.13	0.10	0.04

*AF* average volume flow, *ANF* average negative flow, *APF* average positive flow, *CSAmax* minimum cross-sectional area, *CSAmin* maximum cross-sectional area, *mPAD* main pulmonary artery diameter, *mPADmax* maximum main pulmonary artery diameter, *mPADmin* minimum main pulmonary artery diameter, *mPAP* mean pulmonary artery pressure, *PNV* negative peak velocity, *PPV* positive peak velocity, *RF* regurgitant fraction, *RVEDV* right ventricular end-diastolic volume, *RVEF* right ventricular ejection fraction, *RVMED* right ventricular myocardial mass end-diastolic, *RVMES* right ventricular myocardial mass end-systolic, *RVMI* right ventricular mass index, *RVESV* right ventricular end-systolic volume, *RVSV* right ventricular stroke volume. Data was presented as mean ± SD, and P values were given as COPD versus non-COPD groups

### The volume of distal pulmonary arteries was increased in patients with COPD

Morphometric analysis of pulmonary arteries using Weigert-van Gieson staining and α-SMA-IHC staining showed a thicker vessel wall in COPD lungs compared to non-COPD lungs (Fig. 1b). Semi-quantitative analysis of the morphometric study demonstrated that the WT (Fig. 1c), WT% (Fig. 1d), and WA% (Fig. 1d) were significantly greater in COPD lungs (37.07 ± 18.07 μm, 64.76 ± 19.48%, and 55.38 ± 23.46%, respectively) relative to non-COPD lungs (18.71 ± 3.35 μm, 29.17 ± 5.36%, and 39.67 ± 6.78%, respectively), although the ED value was not statistically different between the COPD and the non-COPD lungs (*P* = 0.687, Fig. 1c).

### A correlations between the cMRI finding and airway limitation in COPD patients

**Fig. 2:**
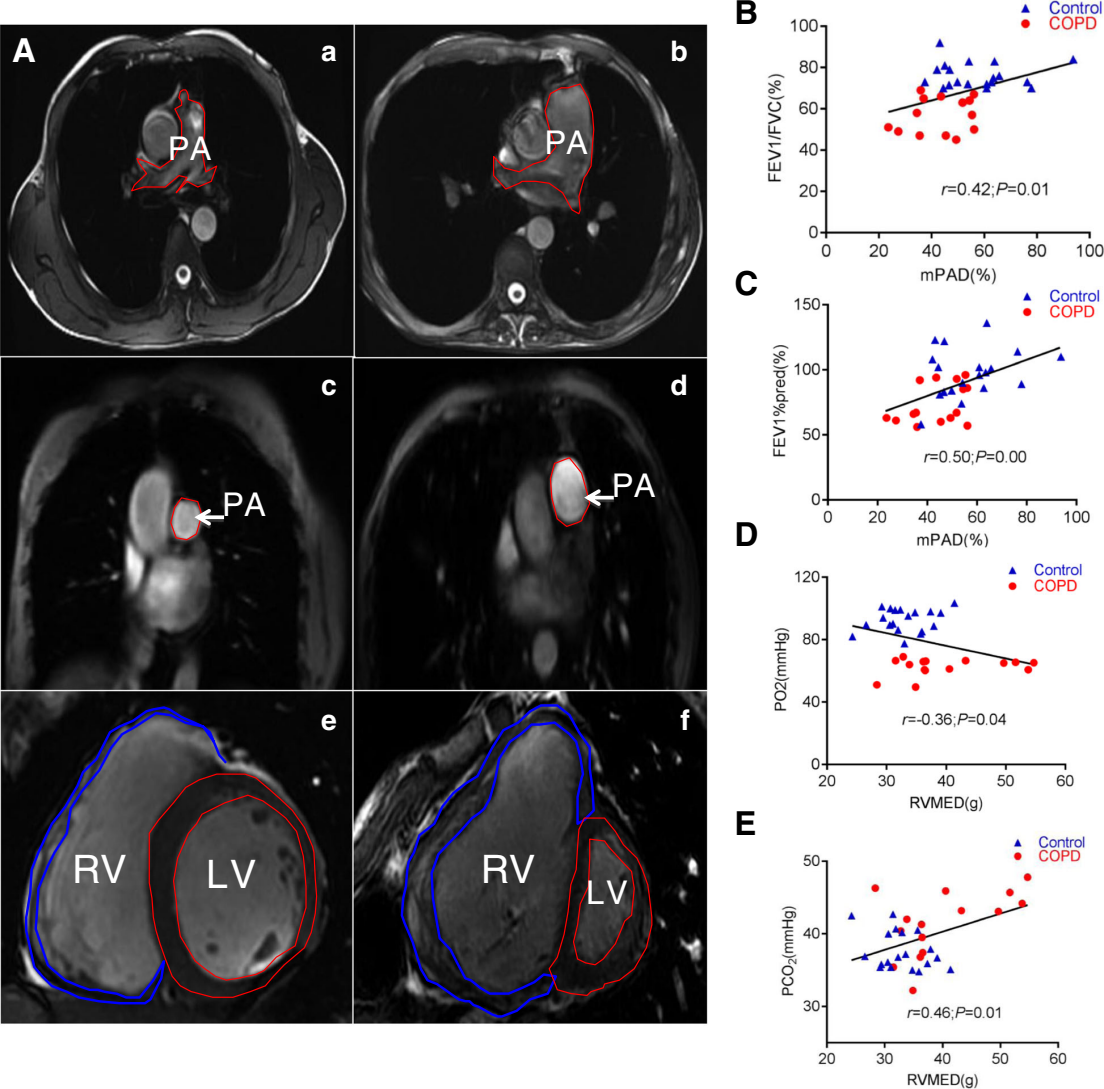
Ventricular dimensions and pulmonary circulation parameters on cMRI. (**a**) Representative images of cMRI movie showed the main pulmonary artery and ventricle. (a) A main pulmonary artery FIESTA short axis image of non-COPD patients; (b) a main pulmonary artery FIESTA short axis image of COPD patients; (c) a main pulmonary artery vertical position image of non-COPD patients; (d) a main pulmonary artery vertical position image of COPD patients; (e) a left and right ventricle short axis image of non-COPD patients; (f) a left and right ventricle short axis image of COPD patients. (**b**-**e)** Correlations between pulmonary artery parameters and pulmonary functions. (**b**) Correlation between mPAD and FEV1/FVC; (**c**) correlation between mPAD and FEV1%pred: (**d**) correlation between RVMED and PO2; (**e**) correlation between RVMED and PCO2. PA: main pulmonary artery; REMED: right ventricular myocardial mass end diastolic; RV: right ventricle; LV: left ventricle; mPAD%: the main pulmonary artery distensibility; PO_2_: arterial partial pressure of oxygen; PCO_2_: partial pressure of carbon dioxide; FEV_1_/FVC: forced expiratory volume in one second/forced vital capacity; FEV1%pred: forced expiratory volume in one second total predicted value

Next, we sought to analyze whether the cMRI finding had a relationship with pulmonary functions in COPD. The correlation analysis showed that the mPAD% was positively correlated with pulmonary function parameters FEV_1_/FVC and FEV_1_%pred, with a correlation coefficient of *r* = 0.42 (*P* < 0.05) (Fig. 2b) and *r* = 0.50 (*P* < 0.01), respectively (Fig. 2c). In addition, RVMED was found to positively correlate with PCO_2_ (*r* = 0.46, *P* < 0.05) (Fig. 2d), but negatively correlate with PO_2_ (*r* = − 0.36, *P* < 0.05) (Fig. 2e).

### Correlations between the volume of distal pulmonary arteries, cMRI findings, and lung functions

**Fig. 3:**
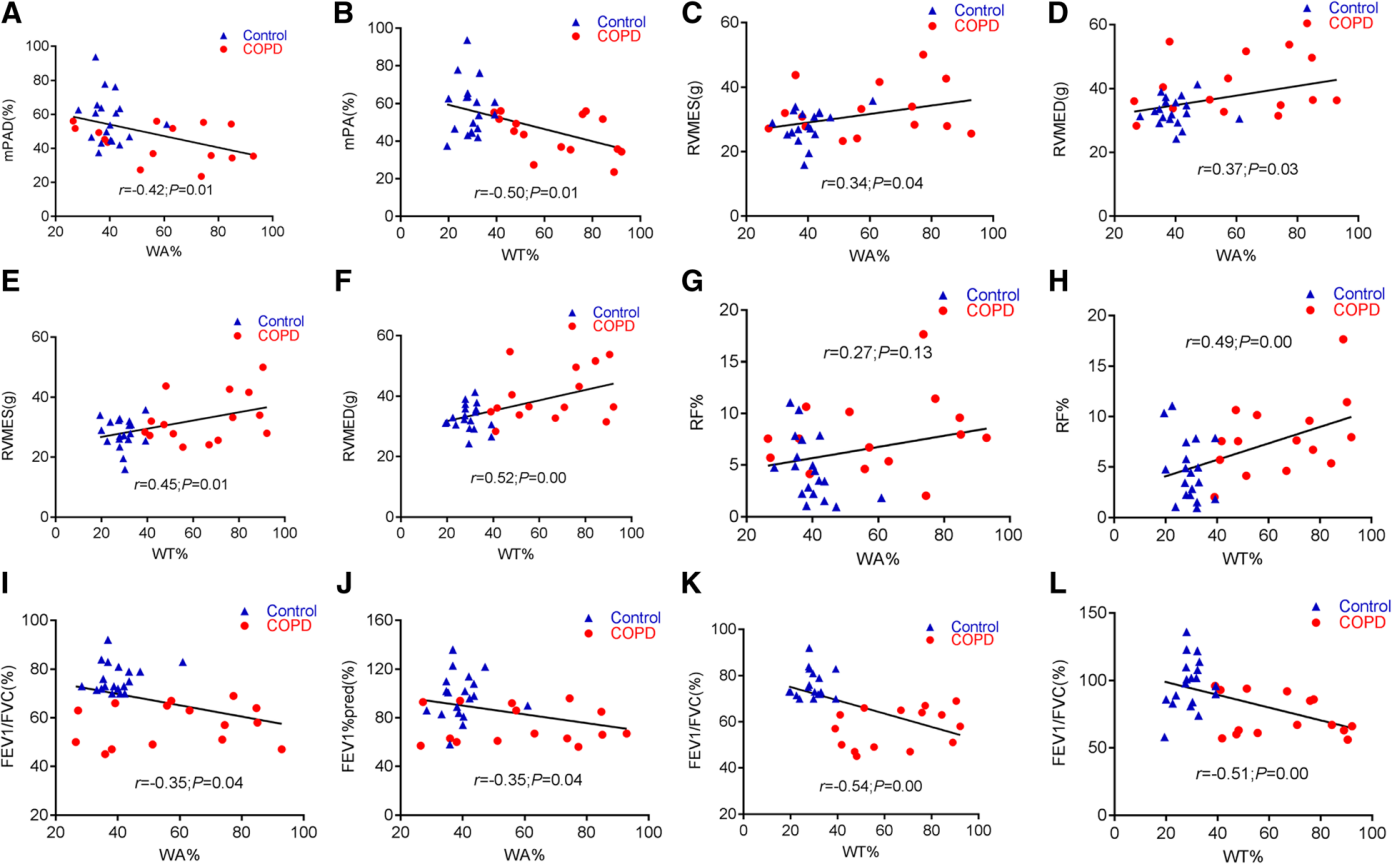
Correlations between the volume of distal pulmonary arteries and cMRI findings and pulmonary functions. **a** The correlation between WA% and mPAD% (*r* = − 0.42,*P* < 0.05); (**b**) the correlation between WT% and mPAD% (*r* = − 0.50,*P* < 0.01); (**c**) the correlation between WA% and RVMES (*r* = 0.34,*P* < 0.05); (**d**) the correlation between WA% and RVMED (*r* = 0.37,*P* < 0.05); (**e**) the correlation between WT% and RVMES (*r* = 0.45,*P* < 0.01); (**f**) the correlation between WT% and RVMED (*r* = 0.52,*P* < 0.01); (**g**) the correlation between WA% and RF% (*r* = 0.27,*P* = 0.13); (**h**) the correlation between WT% and RF (*r* = 0.49,*P* < 0.01); (**i**) the correlation between WA% and FEV_1_/FVC (*r* = − 0.35,*P* < 0.05); (**j**) the correlation between WA% and FEV_1_%pred (*r* = − 0.35,*P* < 0.05); (**k**) the correlation between WT% and FEV1/FVC (*r* = − 0.54, *P* < 0.05); (**l**) the correlation between WT% and FEV1%pred (*r* = − 0.51, *P* < 0.05). FEV_1_/FVC: forced expiratory volume in one second/ forced vital capacity; FEV1%pred: forced expiratory volume in one second total predicted value; mPAD%: the main pulmonary artery distensibility; RF%: regurgitant fraction; RVMED: right ventricular myocardial mass end-diastolic; RVMES: right ventricular myocardial mass end-systolic; WA%: the pulmonary artery smooth muscle area accounted for the percentage of total vascular area; WT%: the pulmonary artery smooth muscle thickness accounted for the percentage of vascular diameter

We next further explored whether the volume of distal pulmonary arteries was correlated with cMRI findings and pulmonary functions in COPD. Indeed, the volume of distal pulmonary arteries was inversely correlated with lung functions (FEV_1_/FVC and FEV_1_%pred) but positively correlated with cMRI findings (mPAD%, RVMES, and RVMED) (Fig. 3). The correlation coefficients between WA% and mPAD, WT% and mPAD%, WA% and FEV_1_/FVC, WA% and FEV_1_%pred, WT% and FEV1/FVC, WT% and FEV1%pred were *r* = − 0.42 (*P* < 0.05) and *r* = − 0.50 (*P* < 0.01), *r* = − 0.35 (*P* < 0.05), *r* = − 0.35 (*P* < 0.05), *r* = − 0.54 (*P* < 0.05), and *r* = − 0.51 (*P* < 0.05), respectively. The respective correlation coefficients between WA% and RVMES, WA% and RVMED, WT% and RVMES, WT% and RVMED, WA% and RF%, WA% and RF% were *r* = 0.34 (*P* < 0.05), *r* = 0.37 (*P* < 0.05), *r* = 0.45 (*P* < 0.01), *r* = 0.52 (*P* < 0.01), *r* = 0.27 (*P* = 0.13) and *r* = 0.49 (*P* < 0.01), respectively (Fig. 3).

### An elevated expression of NOX4 and extracellular matrix deposition in artery smooth muscle (ASM) of distal pulmonary arteries of COPD

**Fig. 4:**
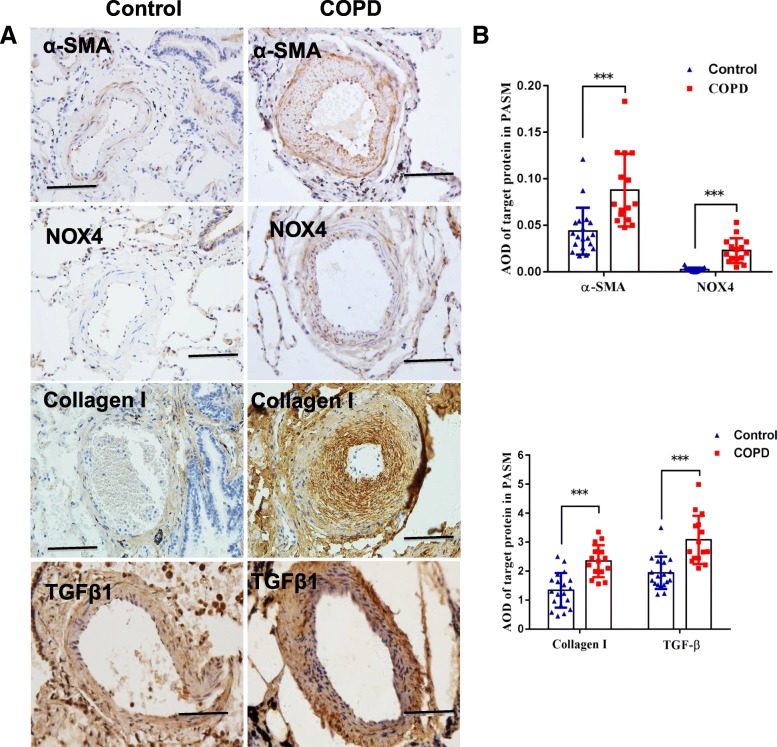
An increased expression of NOX4, α-SMA, collage I and TGFβ1 in distal pulmonary arteries of COPD patients. **a** immunohistochemical (IHC) staining showed an elevated expression of NOX4, and collage I in the smooth muscle of small pulmonary artery of COPD lungs compared to normal control lungs; (**b**) The expression of NOX4 (top panel) and collage I (bottom panel) in non-COPD and COPD lungs determined by absorbance optical density (AOD) values of IHC. Compared to non-COPD lung, *P < 0.05, *** *P* < 0.01 (*N* = 19 for non-COPD; *N* = 15 for COPD). Bars = 100 μm for all images in **a**

**Fig. 5:**
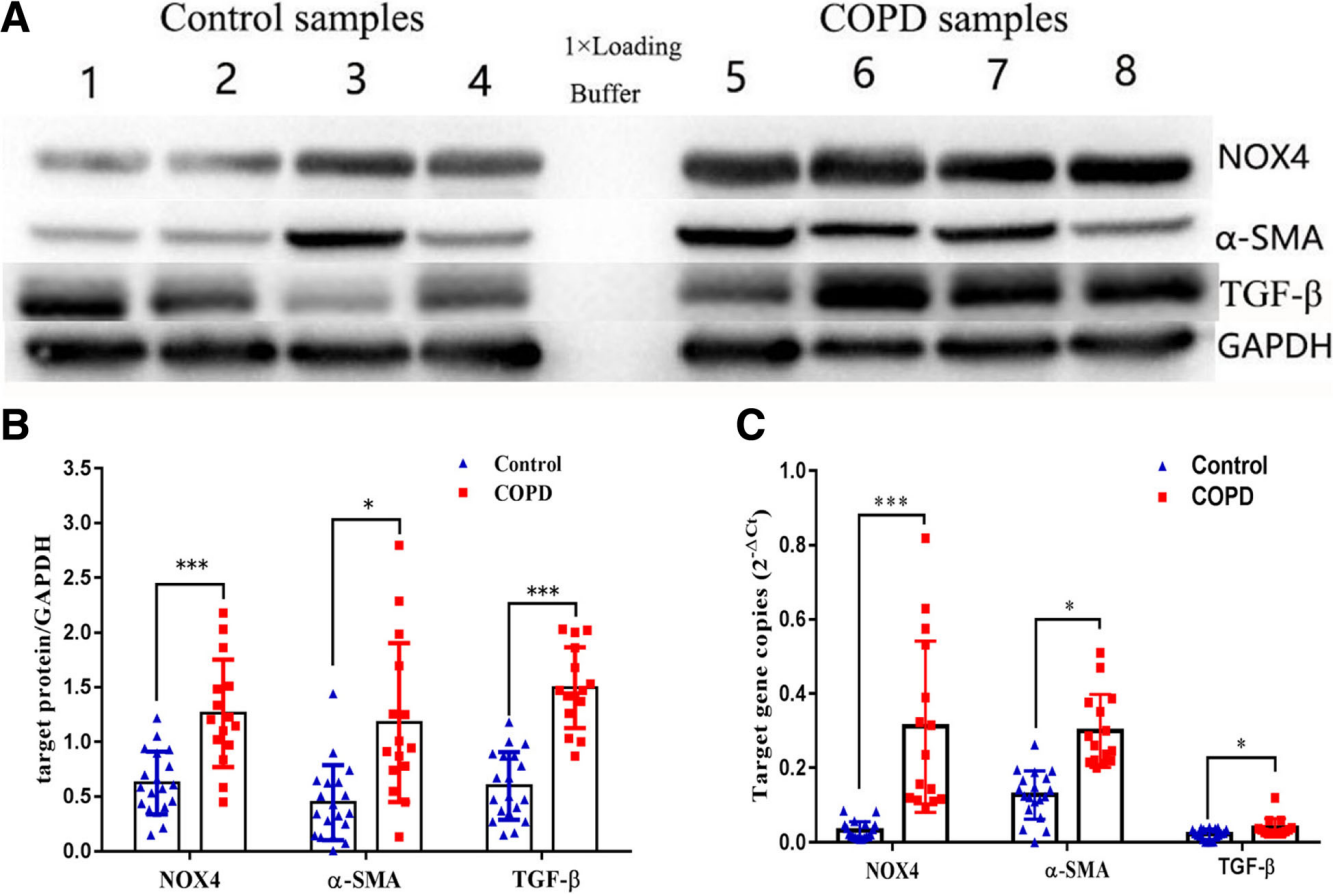
An elevated expression of NOX4, α-SMA and TGFβ1 proteins in human COPD lung tissues. **a** Representative images of immunoblots showed more abundant NOX4, α-SMA and TGF**β**1 proteins in COPD lung specimens as compared with of normal lung controls as determined by an immunoblotting assay; (**b**) Semi-quantitative analysis of the relative expression of NOX4, α-SMA and TGF**β**1 proteins in COPD lungs by densometric analysis of immunoblots; (**c**) Quantitative analysis of the relative expression of NOX4 and α-SMA transcripts in COPD lung by reverse-transcriptional PCR. Compared with normal control lungs, *: *P* < 0.05, ***: *P* < 0.01 (*N* = 19 for non-COPD; *N* = 15 for COPD)

**Fig. 6:**
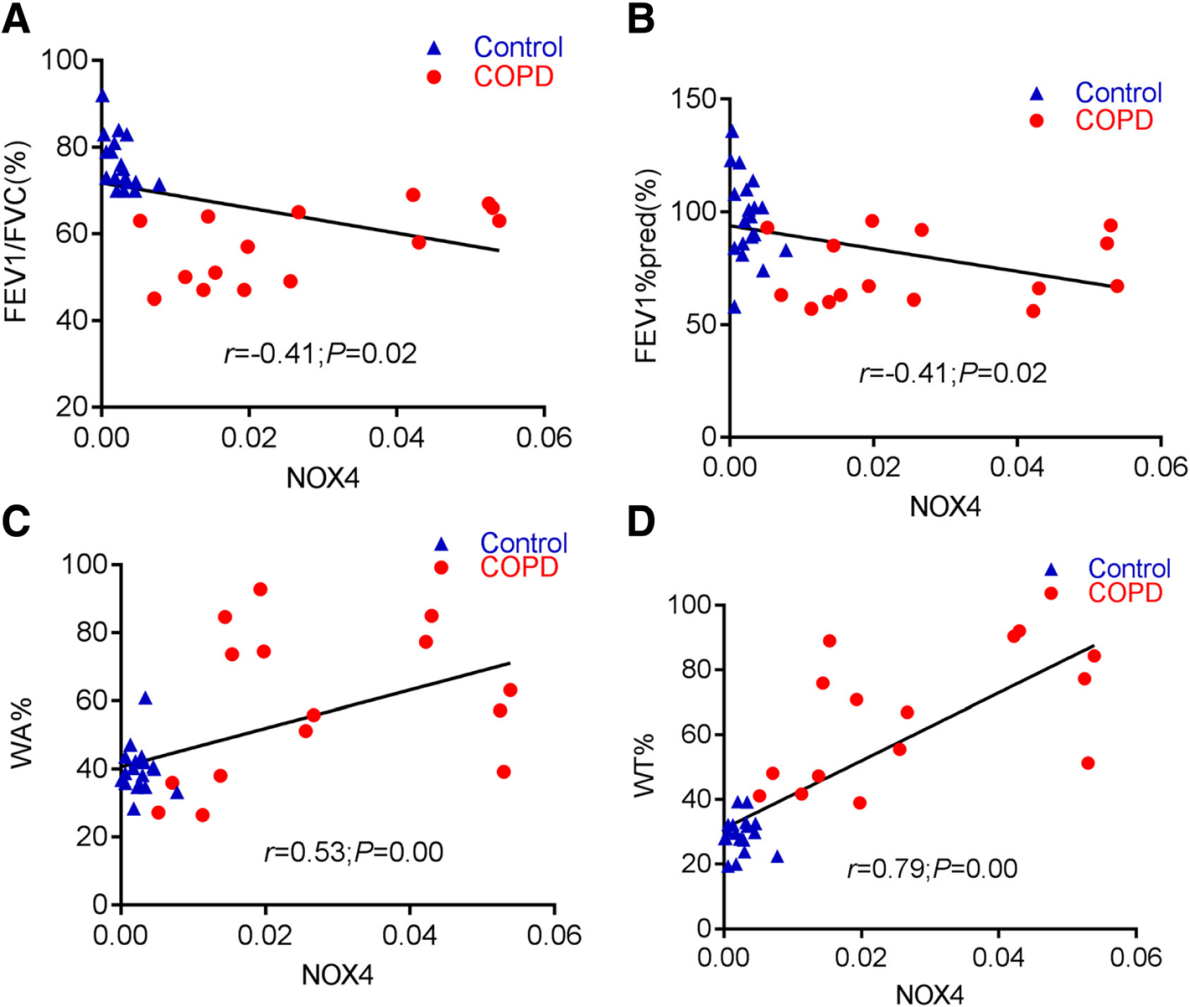
Correlations of the abundance of NOX4 protein in pulmonary distal arteries with the volume of distal pulmonary arteries and pulmonary functions. **a** The correlation between the abundance of NOX4 protein in pulmonary distal arteries and FEV1/FVC (r- = 0.411,*P* < 0.05); (**b**) The correlation between the abundance of NOX4 protein in pulmonary distal arteries and FEV1%pred (*r* = − 0.407,P < 0.05); (**c**) The correlation between the abundance of NOX4 protein in pulmonary distal arteries and WA% (*r* = 0.53,P < 0.01); (**d**) The correlation between the abundance of NOX4 protein in pulmonary distal arteries and WT% (*r* = 0.79,P < 0.01). FEV_1_/FVC: forced expiratory volume in one second/ forced vital capacity; FEV1%pred: forced expiratory volume in one second total predicted value; WA%: the pulmonary artery smooth muscle area accounted for the percentage of total vascular area; WT%: the pulmonary artery smooth muscle thickness accounted for the percentage of vascular diameter

In order to investigate whether NOX4 is involved in distal pulmonary artery remodeling, the abundance of NOX4, α-SMA, TGFβ1 and collagen I proteins and transcripts in pulmonary arteries was determined by IHC, Western blot and qRT-PCR. More abundant NOX4, α-SMA, TGFβ1 and collagen I proteins were detected by IHC (Fig. 4), and Western blotting assay (Fig. 5) in pulmonary arteries or lung tissues of COPD compared with non-COPD, respectively. An increased abundance of NOX4, α-SMA and TGFβ1 transcripts was also observed in COPD lungs relative to non-COPD lungs by the RT-PCR assay (*P* < 0.05) (Fig. 5). Moreover, the correlation analysis further demonstrated that the abundance of NOX4 protein in pulmonary arteries was positively correlated with WA% and WT%, but inversely correlated with pulmonary functions. The correlation coefficients between NOX4 and WA%, NOX4 and WT%, NOX4 and FEV1/FVC, NOX4 and FEV1%pred were *r* = 0.79 (*P* < 0.01), *r* = − 0.41 (*P* < 0.05), *r* = − 0.4 (*P <* 0.05), *r* = 0.53 (*P* < 0.01), respectively (Fig. 6).

### TGFβ1 augmented the expression of NOX4, α-SMA, and collagen I in HPASMCs

**Fig. 7:**
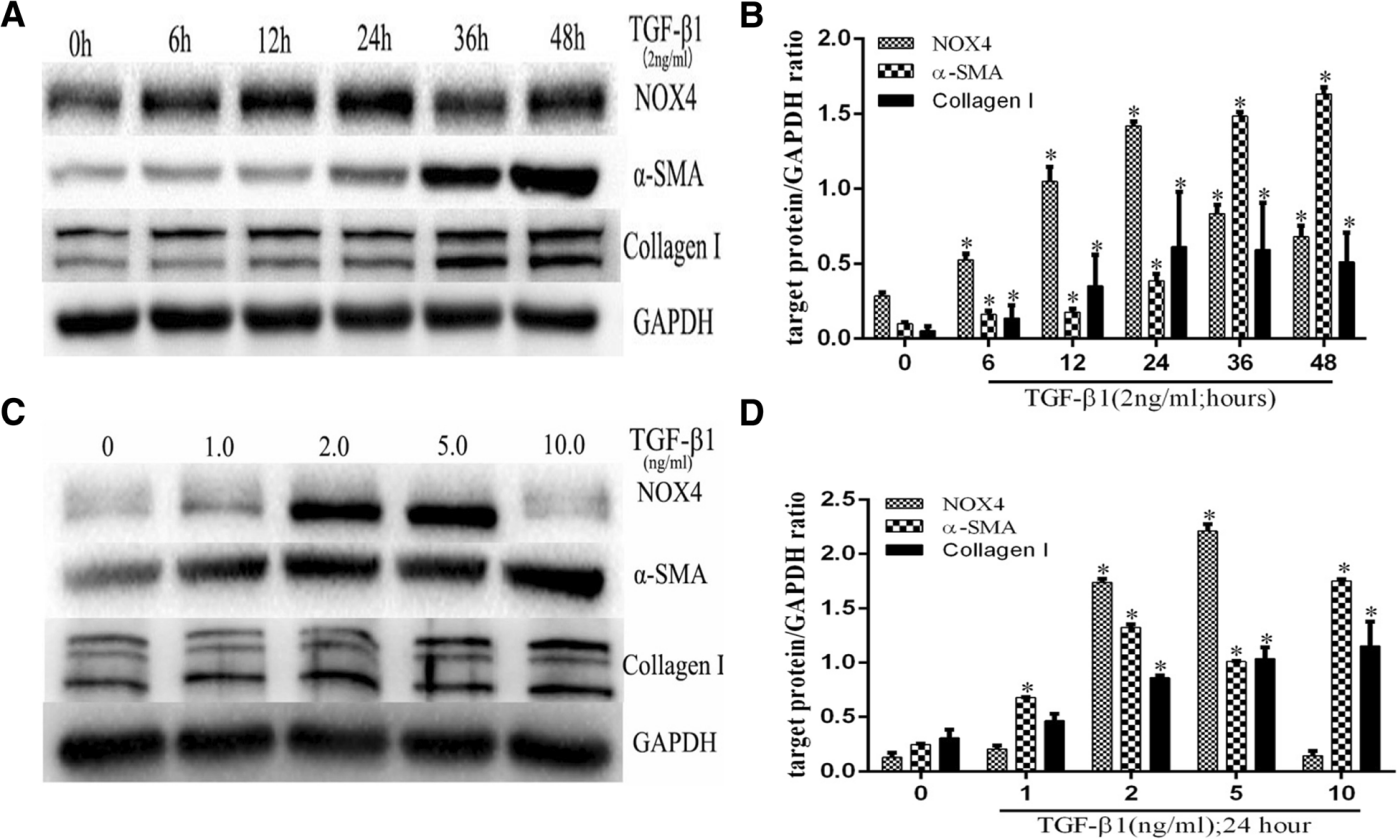
A dynamic expression of NOX4, α-SMA, Collagen I induced by TGFβ1 in human primary artery smooth muscle cells (HPASMCs). HPASMCs were cultured in the presence of TGFβ_1_ at various concentrations (0–10 ng/mL) for different time periods (0–48 h) before the cell lysates were used for analysis. **a** Representative images of immunoblots of NOX4, α-SMA and Collagen I in PASMCs exposed to 2.0 ng/ml of TGFβ1 for indicated time points (0–48 h); (**b**) relative expresions of NOX4, α-SMA and Collagen I determined by a densometric assay of immunoblots of (**a**). **c** Immunoblots of NOX4, α-SMA and Collagen I in PASMCs exposed to indicated concentrations of TGFβ1 (0-10 ng/mL) for 24 h; (**d**) relative expresions of NOX4, α-SMA and Collagen I determined by a densometric assay of immunoblots (**c**). Bar graphs represent mean ± SEM from three independent experiments. Compared with the TGFβ1 untreated group, *: *P* < 0.05 (*N* = 3)

**Fig. 8:**
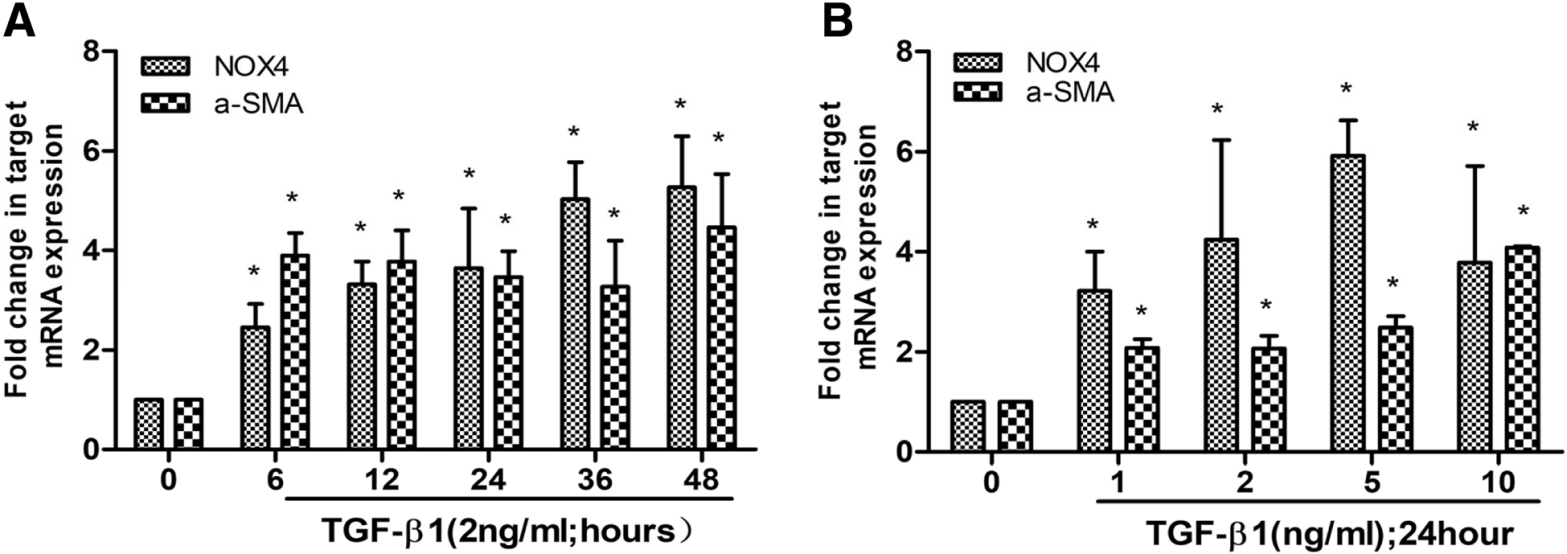
A dynamic induction of NOX4 and α-SMA transcripts by TGF-β1 in human primary artery smooth muscle cells (HPASMCs) determined by RT-PCR assay. HPASMCs were cultured in the presence of TGFβ_1_ at various concentrations (0–10 ng/mL) for different time periods (0–48 h) before the cell total RNA was used for RT-PCR analysis. **a** A dynamic induction of NOX4 and α-SMA transcripts in HPASMCs exposed to 2.0 ng/ml of TGFβ1 at indicated time points; (**b**) a dynamic induction of NOX4 and α-SMA transcripts in HPASMCs exposed to indicated concentration of TGFβ1 for 24 h. Data represent mean ± SEM from three independent experiments. Compared with the TGF-β1 untreated group, *: *P* < 0.05 (*N* = 3)

**Fig. 9:**
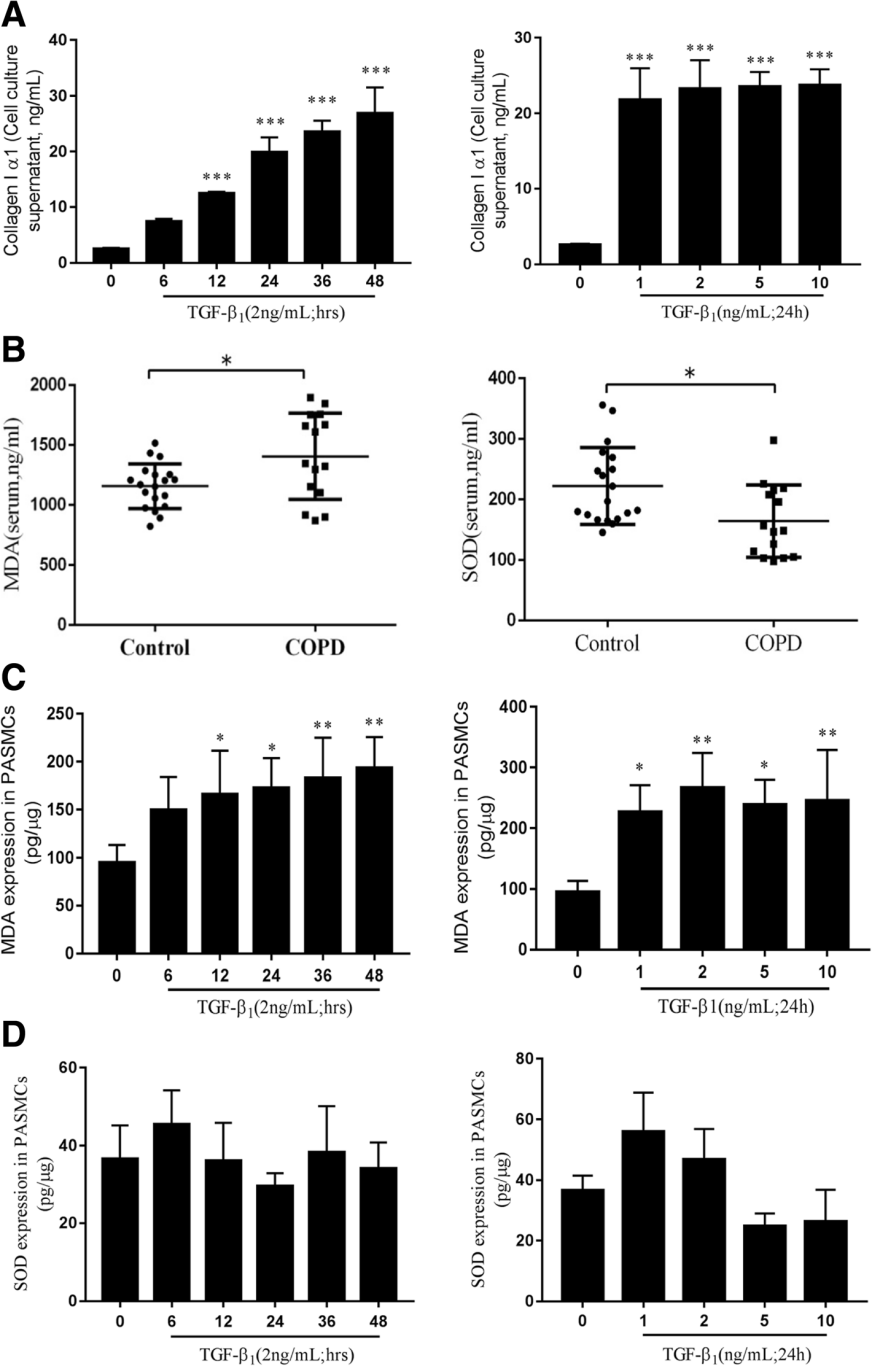
Impacts of TGFβ1 on the production of Collagen type 1 alpha1, malondialdehyde (MDA) and superoxide dismutase (SOD) in human primary artery smooth muscle cells (HPASMCs). HPASMCs were cultured in the presence of TGFβ_1_ at various concentrations (0–10 ng/mL) for different time periods (0–48 h) before the supernatants were used for analysis. **a** The production of Collagen type 1 alpha1 in HPASMCs exposed to 2.0 ng/ml of TGF-β1 for indicated time (left panel) and to indicated concentration of TGFβ1 for 24 h (right panel); (**b**) A higher concentration of MDA (left panel) but a lower concentration of SOD (right panel) in sera of COPD patients relative to healthy subjects; (**c**) the production of MDA (left panel) and SOD (right panel) in HPASMCs exposed to 2.0 ng/ml of TGF-β1 for indicated time; (**d**) the production of MDA (left panel) and SOD (right panel) in HPASMCs exposed to indicated concentration of TGFβ1 for 24 h. Compared to untreated group, *: *P* < 0.05. Bar graphs represent mean ± SEM from three independent experiments. Compared to untreated group, *: *P* < 0.05; **: *P* < 0.01; ***: *P* < 0.001

Previous study has demonstrated that the NOX4 had important implication in pulmonary vascular remodeling and could be induced by TGFβ in HPASMCs [12]. In agreement with this finding, a dynamic induction of NOX4, α-SMA, and collagen I proteins and transcripts of HPASMCs by TGFβ1 was also observed in this study. Significantly more abundant NOX4, α-SMA, and collagen I proteins (Fig. 7) and transcripts (Fig. 8) were induced in HPASMCs by various doses of TGFβ1 for different time periods (*P* < 0.05). Interestingly, the TGFβ1-induced NOX4 expression was dynamic but not in a time- or dose-dependent manner in this cell type, i.e. the most induction of NOX4 was observed in HPASMCs exposed to 2.0–5.0 ng/mL of TGF-β1 at 24 h (Fig. 7), although a time- and dose-dependent induction of α-SMA, and collagen I was found in cells treated with 0.0–10.0 ng/mL of TGFβ1 (Fig. 7). The induction of NOX4, α-SMA, and collagen I proteins in HPASMCs was further confirmed by RT-PCR assay (Fig. 8). Of interest, TGFβ1 also exhibited a capacity to significantly induce the expression of Collagen type 1 alpha1 in HPASMCs as determining their concentrations in supernatants of cell cultures (Fig. 9d).

### TGFβ1 altered the production of MDA, SOD and ROS in HPASMCs

**Fig. 10:**
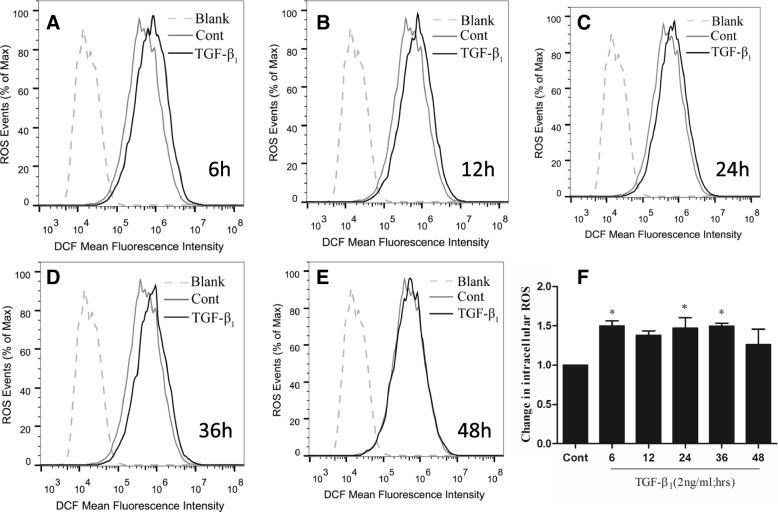
Impacts of TGFβ1 on the production of ROS in human primary artery smooth muscle cells (HPASMCs). HPASMCs were cultured in the presence of TGFβ_1_ at 2 ng/mL for different time periods (0–48 h) before the cells were used for analysis of ROS production by a cytometric assay. **a**-**e** Histogram of flow cytometric analysis using the H2DCF-DA dye to detect enhancement in ROS levels where cell numbers are plotted as a function of the fluorescence intensities at time point of (**a**) 6 h, (**b**) 12 h, (**c**) 24 h. (**d**) 36 h and (**e**) 48 h. (**f**) Bar chart representing the normalized relative ROS levels in the different time point as determined from the same analysis Bar graphs represent mean ± SEM from three experiments. Compared to untreated group, *: *P* < 0.05 (*N* = 3).

To investigate whether the imbalance of oxidant/antioxidants is involved in the development of COPD, contents of oxidants (MDA, lipid hydroperoxide) and antioxidants enzymes (SOD) were evaluated in sera of patients with COPD and non-COPD, and HPASMCs exposed to TGFβ1. As expected, a respective higher and lower concentrations of serum MDA and SOD were determined in the sera of COPD patients as compared with those of non-COPD individuals (*P* < 0.05) (Fig. 9b). In consistent with above result of sera of COPD patients, TGFβ1 also exhibited a capacity to significantly induce HPASMCs produce MDA, but have no effect on SOD production as determining their concentrations in HPASMC or supernatants of cell cultures (Fig. 9c and d). In addition, the effect of TGFβ1 on the production MDA in HPASMCs was in a time- and dose-dependent manner (Fig. 9c and d). In addition, the exposure of HPASMCs to TGFβ1 led an increased ROS production in vitro (Fig. 10), suggesting the involvement of imbalance of oxidant-antioxidants in HPASMC remodeling. These results clearly suggest an implication of TGFβ1/NOX4-mediated ROS production and oxidant/antioxidant imbalance in the development of COPD and distal pulmonary artery remodeling.

## Discussion

In the present study, morphological changes of pulmonary arteries and pulmonary blood flow, and the expression of NOX4 in distal pulmonary arteries in COPD lungs were investigated. The results demonstrated an involvement of NOX4, the imbalance of oxidant/antioxidants in pulmonary vascular remodeling and pulmonary functions in COPD patients. Mechanistically, TGFβ may contribute to induction of NOX4 and production of ROS in COPD lungs and human pulmonary arterial smooth muscle cells, suggesting the occurrence of distal pulmonary vascular remodeling during the early stage of COPD development. The remodeling of distal pulmonary vessels may affected the morphology and function of pulmonary artery and RV, and become a main cause of PAH in patients with severe COPD.

The pathogenic characteristic of COPD is a chronic inflammatory damage of airway and/or alveoli, which gradually leads to airway obstruction and alveolar structure disruption and the reduction of gas exchange. Moreover, the chronic hypoxia and persistent oxidative damage result in alveolar hypoxia and pulmonary vascular remodeling, thereby promoting pulmonary vascular resistance (PVR) and the increase of pulmonary arterial pressure in patients with COPD, which eventually may lead to the development of PH, an important negative prognostic factor in COPD patients [41, 42]. The 5-year survival rate of patients with COPD having mPAP> 25 mmHg is significantly lower compared to patients without PH [43, 44]. Previous studies found that the mPAP in stable COPD patients was usually 25–30 mmHg, which was significantly higher than that in patients with other chronic lung diseases [44, 45]. Therefore, an early diagnosis and intervention of COPD with PH is of paramount importance.

Pathologically, the abnormal proliferation of smooth muscle and extracellular matrix (ECM) deposition is a key part of the initiation and development of pulmonary vascular remodeling [46, 47]. Experimentally, mice exposed to chronic hypoxia showed a moderate membrane thickening and extracellular matrix deposition in pulmonary arteries (50-200 μm), along with a significantly increased WA% and WT% of pulmonary arteriole, RV hypertrophy index and RV systolic pressure, compared with mice exposed to normoxia [47]. In the present study, values of WT, WT%, and WA% of pulmonary arterioles in patients with GOLD grades 1 and 2 of COPD were significantly greater than those in non-COPD group. In addition, the pulmonary arteriolar smooth muscle marker α-SMA was augmented in distal pulmonary of COPD lungs relative to non-COPD lungs. The correlation analysis further demonstrated that both WA% and WT% in pulmonary arterioles were inversely correlated with parameters of pulmonary function of FEV_1_/FVC and FEV_1_%pred in these patients. These results clearly evidenced a pulmonary arteriolar remodeling that characterized by hyperplasia and smooth muscle thickening was developed, which was correlated with the obstruction degree of COPD airflow in patients with grade 1 and 2 COPD.

cMRI is a gold standard for measuring cardiac function, confirmed by extensive clinical trials [48, 49], which has been used to replace invasive angiography for the initial evaluation of COPD secondary pulmonary heart disease [50]. In this regard, previous studies revealed that the cMRI-measured mPAD% was a good index for evaluating the elasticity of wall of pulmonary arteries [51, 52]. In the present study, the cMRI-measured mPAD% was significantly lower in COPD patients compared with non-COPD individuals. In consistence, the mPAD% was positively correlated with parameters of pulmonary function in patients with COPD (FEV1/FVC and FEV1%pred). While the index of WA% and WT%, remodeling of pulmonary arterioles was inversely correlated with cMRI-measured mPAD%. Results in this study also showed that pulmonary vascular changes characterized by remodeling of distal pulmonary arteriolar smooth muscles and decreased dilatation of proximal main pulmonary arteries might have already occurred in COPD lungs before the definite manifestation of PH. Furthermore, the pulmonary vascular remodeling in COPD was related to the severity of airflow obstruction. These findings further indicate the existence of a common mechanism involved in pulmonary vascular remodeling and ASM remodeling in COPD lungs.

Alterations in the structure and function of RV usually occur in COPD and are characterized by ventricular dilatation and cardiac hypertrophy. These changes of myocardium are adaptive to accommodate an increased pulmonary arterial pressure, and result in the impaired ventricular function, low cardiac output, and heart failure [53]. cMRI could be used for evaluating right ventricular functions, including RVMED, RVMES, RVMI, ANF, and RF% in patients with COPD. Of note, the aforementioned cMRI parameters were significantly increased in COPD patients in this study. Moreover, the value of RVMES, RVMED, and RF% was correlated with remodeling indexes of distal pulmonary arterioles such as WA% and WT%. These changes of pulmonary vessels and right ventricular morphology imply that the remodeling of distal pulmonary arteriolar smooth muscle and decreased dilatation of the proximal main pulmonary arteries may occur in COPD before a definite PH can be determined. In addition, such a structural change of distal pulmonary arterioles in patients with COPD may subsequently affect changes in the main pulmonary artery blood rheology, as well as the morphology and function of right ventricle.

**Fig. 11:**
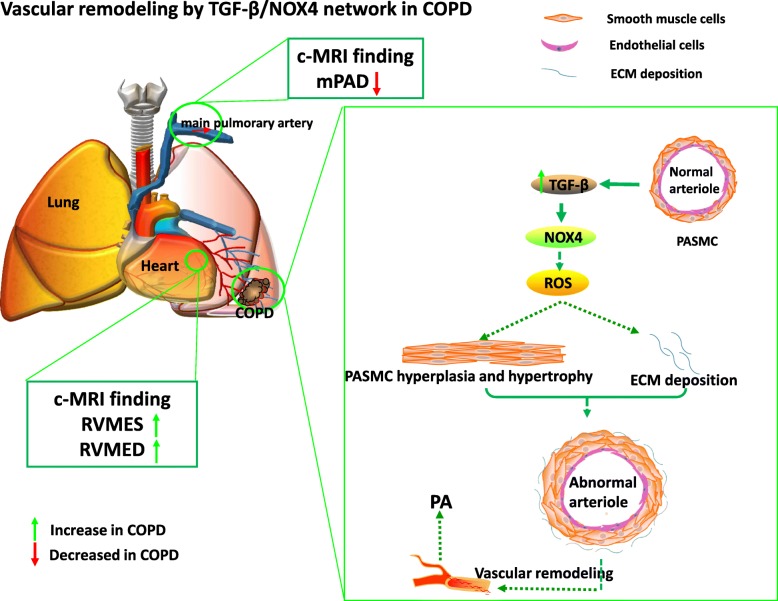
A possible mechanism of distal pulmonary arteriolar remodeling and its role in pathogenesis of PH in COPD. The scheme illustrates the possible mechanism of NOX4 in distal pulmonary arteriolar remodeling and pathogenesis of PH in COPD. In an early stage of COPD, distal arteriolar remodeling distal arteriolar remodeling and changes in proximal pulmonary artery blood rheology, structure and function of the right heart, including the decreased mPAD, and increased RVMES and RVMED could be determined by cMRI before a definite PH in COPD patients. Pathogenically, the hyperactivated TGFβ signaling triggered the expression of NOX genes, particularly the NOX4 gene in PASMCs and endothelial cells of distal vascules, and smooth muscle cells and epithelial cells of distal airways in COPD lungs. The augmented expression of NOX4 could induce the production of ROS, which in turn led the hyperplasia and hypertrophy of PASMCs, and ECM deposition. As a consequence, these resulted in the distal pulmonary vascular remodeling, and eventually led PAH in patients with a late stage of COPD. Solid lines indicate previously confirmed mechanisms, and dished lines represent proposed mechanisms that need further investigations. mPAD: main pulmonary artery diameter; PAH: pulmonary arterial hypertension; RVMED: right ventricular myocardial mass end-diastolic; RVMES: right ventricular myocardial mass end-systolic

Oxidative stresses have an important implication in the development and progression of the pulmonary disease-related hypertension. NADPH oxidases (NOXs) are an important source of intracellular ROS production, which recently gains an increased interest in the pathogenesis of COPD and PH. Several lines of evidence showed that an activation of NOX was an important mechanism in the pathogenesis of hypoxia-induced PH in mice [16, 17], in which the NOX-produced ROS participated in the development of chronic hypoxic pulmonary vascular remodeling [54]. In this regard, ROS has widely been recognized as an inducer of vascular wall cell proliferation and vasoconstriction [18]. Therefore, targeting mitochondria-derived ROS production may offer particularly effective in preventing hypoxia-induced PH [9, 55, 56]. Indeed, an inhibition of NOX/vascular peroxidase 1 (VPO1) pathway and inflammatory reaction showed a possibility to prevent cardiovascular remodeling in the hypoxia-induced pulmonary hypertensive rat model [57]. This notion was supported by evidences of that the NOX4 inhibitor GKT137831 could attenuate hypoxia-induced pulmonary vascular cell proliferation [58], and the cyclic stretch induced mitochondrial ROS and NOX4 signaling in PASMCs [59]. Consistently, more abundant NOX4, α-SMA, and collagen I proteins were observed in smooth muscles of distal pulmonary artery in patients with COPD than non-COPD subjects in present study. Correlation analysis further showed that the NOX4 expression was inversely correlated with pulmonary functions, but were positively correlated with remodeling indexes of pulmonary arterioles, such as WA% and WT%. Mechanistically, TGFβ1 might be a main inducer of the intracellular ROS production by up-regulating NOX4 in arteriolar smooth muscle cells, suggesting that TGFβ-induced NOX4 promotes cell transdifferentiation and ECM deposition (α-SMA and collagen I). These further indicate an involvement of NOX4 in the development of pulmonary artery remodeling and PAH in COPD (Fig. 11).

The imbalance of oxidant/antioxidants recently receives an increasing attention in the pathogenesis of COPD and PH. In this regard, MDA is a product of lipid peroxidation and marker of oxidative stress in COPD. On the other hand, SOD is an important antioxidant enzyme, which can effectively eliminate superoxide anion radical generated in biological oxidation process and balance oxygen radicals in the body. A number of studies showed that the MDA level was elevated in sera [60] and plasma [61] of patients with COPD. The increased MDA level might correlate to oxidative stress such as cigarette smoke-induced degeneration of unsaturated fatty acids on the cell membrane [62, 63]. However, the involvement of SOD activity in patients with COPD was inconsistent [60, 64]. In the present study a significant higher concentration of serum MDA was found in COPD patients relative to non-COPD individuals, but only moderate alteration of SOD level was observed between sera of COPD and non-COPD patients. In vitro study also demonstrated that TGFβ1 could induce the production of MDA and ROS, but suppress SOD production in HPASMCs. Therefore, it may be speculated that SOD is produced in response to an early stage of oxidative stress, whereas a long-term oxidative damage leads to the consumption of its antioxidant capacity in patients with COPD. This notion may suggest that oxidative stresses are a main pathogenic factor in pulmonary vascular remodeling of COPD.

## Conclusion

In conclusion, two main findings were demonstrated in this study (1) distal arteriolar remodeling and changes in proximal pulmonary artery blood rheology, structure and function of the right heart might occur before a definite PH in COPD patients [65]; (2) TGFβ-triggered NADPH oxidase-ROS signaling cascade, particularly the NOX4/ROS signaling was involved in the development of pulmonary arteriole remodeling and PAH in COPD (Fig. 11).

## Abbreviations

6MWD: 6-min walk distance
cMRI: Cardiac magnetic resonance imaging
COPD: Chronic obstructive pulmonary disease
ECM: Extracellular matrix
ED: External diameter of blood vessels
ELISA: Enzyme-linked immunosorbent assay
GOLD: Global Initiative on Obstructive Lung Disease
HE: Hematoxylin and eosin
HPASMCs: Human pulmonary artery smooth muscle cells
HPH: Hypoxic pulmonary hypertension
IPP6.0: Image-Pro Plus 6.0
MDA: Malondialdehyde
mPAD%: Main pulmonary artery distensibility
ROS: Reactive oxygen species
RT-qPCR: Reverse-transcriptional quantitative PCR
RV: Right ventricle
RVEF: Right ventricular ejection fraction
RVMED: Right ventricular myocardial mass end-diastolic
RVMES: Right ventricular myocardial mass end-systolic
RVMI: Right ventricular mass index
RVMM: Right ventricular myocardial mass
SOD: Superoxide dismutase
VOP1: Vascular peroxidase 1
WA%: Pulmonary artery smooth muscle area accounting for the percentage of total vascular area
WT: Wall thickness of pulmonary artery smooth muscle)
WT%: Pulmonary artery smooth muscle thickness accounting for the percentage of vascular diameter).
